# Innovative Biomaterials for Modulating Neuroinflammation and Promoting Repair After Traumatic Brain Injury

**DOI:** 10.3390/pharmaceutics18040477

**Published:** 2026-04-13

**Authors:** Ziwei Wang, Wenlong Yuan, Jin Li, Meng Qin

**Affiliations:** 1 Mental Health Center and Center for Preclinical Safety Evaluation of Drugs, West China Hospital, Sichuan University, Chengdu 610041, China; wangziwei2027@163.com (Z.W.); yuan1398788093@163.com (W.Y.); 2 Department of Clinical Medicine, School of Medicine, University of Electronic Science and Technology of China, Chengdu 610054, China

**Keywords:** traumatic brain injury, neuroinflammation, biomaterials, exosome delivery systems, intelligent materials

## Abstract

Traumatic brain injury (TBI) represents a significant global health challenge with limited effective treatments. The secondary injury phase, characterized by persistent neuroinflammation, is a major contributor to long-term neurological deficits. Conventional therapies face substantial hurdles, including the blood–brain barrier (BBB), short therapeutic windows, and poor neuroregenerative capacity. Innovative biomaterials offer a promising platform to overcome these limitations by providing localized Drug Deliv., immunomodulation, and structural support for neural regeneration. This review outlines the pathological mechanisms of neuroinflammation and repair obstacles following TBI. It then systematically categorizes and discusses the mechanisms of various biomaterials—including natural, synthetic, nano-scale, composite, and intelligent materials—in modulating neuroinflammation. Furthermore, we elaborate on strategies for promoting neural repair, such as constructing regenerative scaffolds, delivering therapeutic agents (e.g., neurotrophic factors, stem cells, and exosomes), and remodeling the regenerative microenvironment. Special emphasis is placed on the emerging application of exosome delivery systems. Finally, we address the challenges in clinical translation and present future perspectives on smart materials, multi-modal systems, and personalized therapies, highlighting the transformative potential of biomaterials in TBI management.

## 1. Introduction

**Figure 1:**
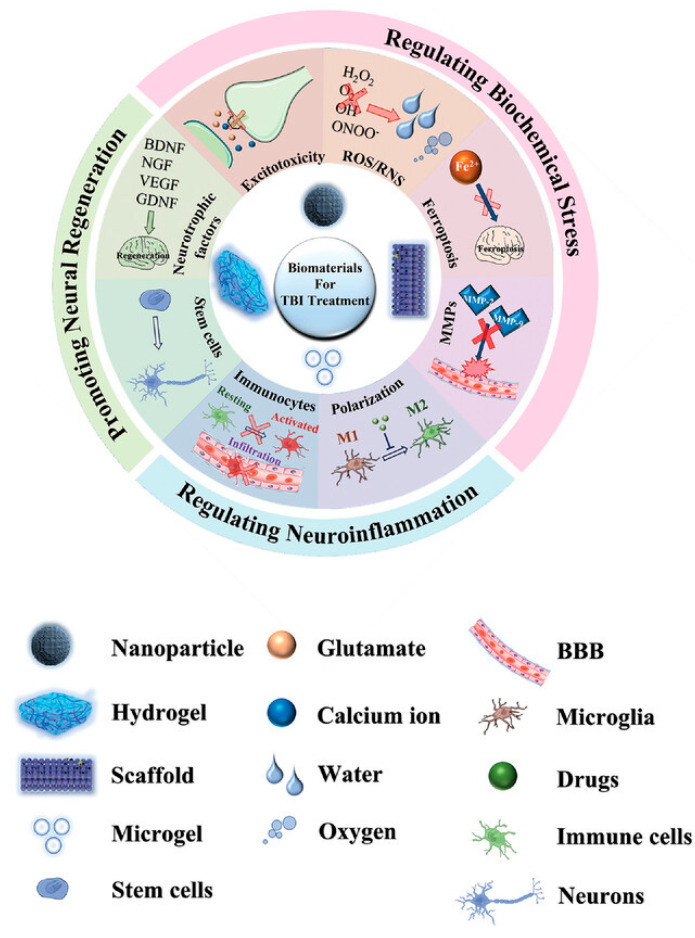
Schematic illustration of microenvironment-regulating biomaterials in TBI [14]. Copyright (2023) Wiley.

Traumatic brain injury (TBI) remains a leading cause of mortality and long-term disability globally, imposing substantial socio-economic burdens on healthcare systems and society [1,2,3,4,5]. The pathology of TBI unfolds through a two-stage process: an initial primary injury involving immediate mechanical damage to brain tissue, followed by a complex and protracted secondary injury phase characterized by cascading molecular events, with neuroinflammation serving as a central driver of ongoing damage [6,7,8]. Traditional therapeutic interventions, including pharmacologic agents, are significantly limited by their inability to effectively cross the blood–brain barrier (BBB), their transient therapeutic effects, and their general lack of capacity to actively promote neural repair and regeneration [9]. In this context, innovative biomaterial platforms present distinct advantages, offering the potential to modulate the hostile local microenvironment, enable precise spatiotemporal delivery of therapeutic agents, and provide critical structural scaffolds to support tissue reconstruction (Figure 1) [10,11,12,13,14]. The purpose of this review is to summarize recent advances in biomaterial-based strategies for controlling neuroinflammation and promoting brain tissue repair after TBI, with a specific focus on the emerging and promising application of exosome delivery systems as a key regenerative tool [15,16,17].

## 2. Mechanisms of Neuroinflammation and Repairing Obstacles After TBI

**Figure 2:**
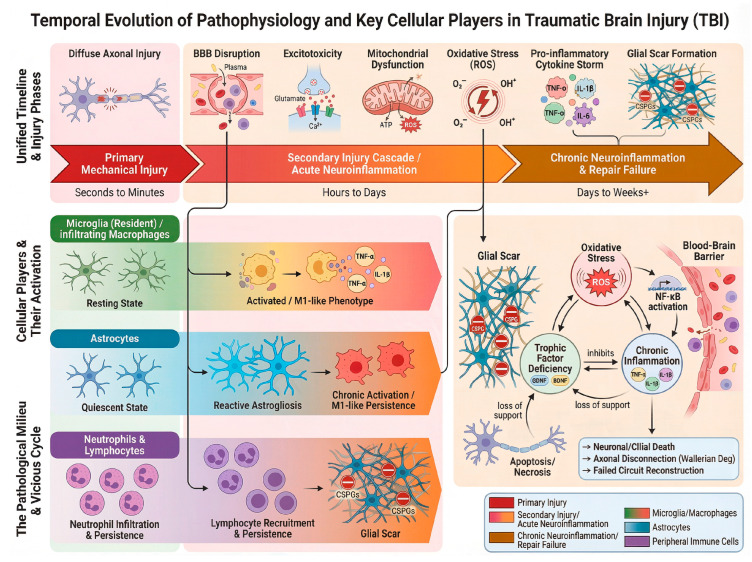
Temporal evolution of pathophysiology and key cellular players in TBI.

Following the initial physical trauma, TBI pathogenesis enters a complex and dynamic secondary phase, characterized by a maladaptive interplay between persistent inflammatory processes and failed repair mechanisms (Figure 2). This chapter details this dynamic cascade, its key cellular orchestrators, the resultant pathological milieu, and the multifaceted barriers that ultimately impede neural repair and functional restoration.

### 2.1. Dynamic Nature of Neuroinflammation

The post-traumatic neuroinflammatory response is not a singular event but a temporally evolving cascade. Its failure to resolve, rather than its initiation, constitutes a primary obstacle to repair and a key driver of long-term neurological deficits. This response unfolds in two critical, opposing phases [18,19].

During the acute phase (hours to days post-injury), the rapid activation of resident microglia and the disruption of the blood–brain barrier (BBB) are pivotal early events [20,21,22,23,24,25]. This facilitates the influx of peripheral immune cells and mediators, a process essential for containing the lesion, phagocytosing debris, and initiating protective signaling [26,27]. This controlled inflammatory response is, therefore, a necessary component of innate CNS defense.

However, in many cases, this response progresses into a persistent chronic phase (weeks to years), transitioning from protective to profoundly detrimental. A sustained release of cytotoxic factors, including reactive oxygen species (ROS) and pro-inflammatory cytokines such as TNF-α and IL-1β, directly exacerbates secondary neuronal apoptosis and synaptic dysfunction [28,29,30]. Concurrently, reactive astrocytes undergo hypertrophy and proliferation, forming a dense glial scar. This scar, while initially sealing the injury site, later acts as a potent physical and chemical barrier to regeneration, largely due to its high content of inhibitory chondroitin sulfate proteoglycans (CSPGs) [31,32,33].

This dynamic duality—where the acute response is necessary but its chronic perpetuation is destructive—explains the failure of historical therapeutic approaches that sought to blunt inflammation indiscriminately. It underscores the critical need for spatiotemporally precise modulation, a task for which advanced biomaterial platforms are uniquely suited, as they can be engineered to intervene in specific pathways at defined time points post-injury [34,35,36].

### 2.2. Key Cellular Players

The pathological progression of TBI is orchestrated by a dynamic interplay between resident central nervous system (CNS) cells and infiltrating peripheral immune cells. Their coordinated, yet frequently dysregulated, interactions create a microenvironment that evolves from initially protective to chronically pathological, fundamentally dictating the balance between recovery and persistent dysfunction.

As the CNS’s primary resident immune cells, microglia are rapid first responders to injury. Their activation initiates crucial early processes such as debris clearance and the release of trophic factors. The historical M1/M2 polarization paradigm, which categorizes microglia into pro-inflammatory (M1, releasing TNF-α, IL-1β, ROS) and anti-inflammatory/reparative (M2) states, provides a useful conceptual framework [24,25,37]. However, this binary model is a significant oversimplification of a more complex in vivo reality, where microglia exist along a dynamic and multidimensional functional continuum [3,21]. It is important to note that while the simplistic M1/M2 dichotomy is contested and does not fully capture the spectrum of microglial phenotypes in vivo, this terminology remains entrenched in the primary experimental literature cited throughout this review. Therefore, to accurately reflect the nomenclature used in the foundational studies describing biomaterial mechanisms, we will retain the terms “M1-like” and “M2-like” or “pro-inflammatory” and “anti-inflammatory/reparative” phenotypes where appropriate. Their use herein should be understood as referring to these broad functional poles—enhancing detrimental inflammation versus promoting resolution and repair—rather than definitive, binary states. The ultimate therapeutic goal is the precise modulation of microglial responses across this continuum to favor a repair-promoting milieu. While their acute activity is essential, sustained microglial activation often shifts toward a chronic pro-inflammatory state that exacerbates neuronal death, synaptic loss, and blood–brain barrier (BBB) disruption [21,22]. This highlights the central therapeutic challenge: achieving precise modulation of microglial responses to preserve their beneficial functions while curtailing their chronic toxic effects, moving beyond the simplistic goal of inducing an M1-to-M2 phenotypic switch.

Reactive astrogliosis is another hallmark of TBI, wherein astrocytes undergo morphological changes and proliferate to form a dense glial scar around the lesion [38,39,40]. This scar epitomizes the dual nature of neuroinflammation. In the acute phase, it serves a vital protective role by helping to restore BBB integrity and isolate the injury core. In the chronic phase, however, it transforms into a major mechanical and chemical barrier to axonal regeneration [41,42]. Reactive astrocytes markedly upregulate the production of inhibitory chondroitin sulfate proteoglycans (CSPGs), such as neurocan and phosphacan. These CSPGs actively repel growing axons and induce growth cone collapse through specific cell surface receptors and subsequent activation of the intracellular Rho/ROCK signaling pathway [31,32,33,38,39]. Thus, therapeutic strategies must aim to temporally manage astrocytic reactivity, mitigating its chronic inhibitory effects without impairing its acute protective functions.

The disruption of the BBB opens a critical gateway for peripheral immune cells, which dramatically amplify the local inflammatory response. Neutrophils are the first to infiltrate, peaking within 24 h. They release matrix metalloproteinases (e.g., MMP-9) that further degrade the BBB, alongside reactive oxygen species and pro-inflammatory cytokines [26,43,44,45,46]. They are followed by monocytes that differentiate into macrophages within the brain parenchyma. These macrophages exhibit considerable plasticity, often augmenting the pro-inflammatory milieu but also potentially contributing to later repair phases [21,47]. The subsequent recruitment of T and B lymphocytes introduces adaptive immune specificity, which may sustain chronic inflammation and link the initial trauma to prolonged immune dysregulation [36,48,49].

In summary, the sequelae of TBI are driven by a dysregulated cellular symphony. The crosstalk between persistently activated microglia, scar-forming astrocytes, and infiltrating leukocytes creates a self-perpetuating cycle of inflammation and inhibition. This intricate synergy presents a formidable challenge but also provides a suite of specific cellular and molecular targets for innovative biomaterial-based interventions designed to rebalance the immune environment and enable repair [50,51,52].

### 2.3. Pathological Microenvironment

The dysregulated cellular responses following TBI coalesce to establish a profoundly inhibitory pathological microenvironment. This milieu is not merely a list of independent hallmarks but a self-reinforcing triad of oxidative stress, chronic inflammation, and trophic factor deficiency, which synergistically suppresses endogenous repair programs [53,54].

A primary and sustained hallmark is the excessive production of reactive oxygen species (ROS), leading to pervasive oxidative stress. This inflicts direct damage on cellular components but also acts as a critical signaling molecule that perpetuates pro-inflammatory transcription factors such as NF-κB, thereby creating a vicious cycle that amplifies both damage and inflammatory signaling. This oxidative environment is fueled by a persistent surge of specific pro-inflammatory cytokines. Tumor necrosis factor-alpha (TNF-α) and interleukin-1 beta (IL-1β) are particularly detrimental, driving not only further inflammatory gene expression but also directly mediating neuronal excitotoxicity and apoptosis through distinct receptor-mediated pathways [20,50,55,56,57].

Concurrently, a critical deficit in essential neurotrophic support, including brain-derived neurotrophic factor (BDNF) and glial cell line-derived neurotrophic factor (GDNF), creates a state of trophic insufficiency [47,49,58,59]. This deprives vulnerable neurons and potential progenitor cells of crucial survival and plasticity signals. These elements interact in a synergistic, maladaptive manner: ROS and pro-inflammatory cytokines can inhibit the expression and signaling of neurotrophic factors, while the lack of trophic support reduces neuronal resilience to oxidative and inflammatory insults. This integrated pathophysiology actively suppresses axonal sprouting, promotes the consolidation of the inhibitory glial scar, and explains the limited success of clinical therapies targeting any single pathway in isolation. It thus provides a compelling rationale for combinatorial biomaterial strategies designed to concurrently quench oxidative stress, modulate specific inflammatory signals, and provide sustained trophic support to reverse this hostile milieu [60,61,62].

### 2.4. Repairing Obstacles

The sustained pathological milieu after TBI initiates a sequence of interlinked pathophysiological events that collectively obstruct functional recovery. The cytotoxic environment, characterized by a persistent surge of specific mediators like TNF-α and the overproduction of reactive oxygen species, directly triggers extensive apoptotic and necrotic death among vulnerable neuronal populations and myelinating oligodendrocytes, thereby depleting the essential cellular substrate for repair [63,64,65,66,67].

Concurrently, this environment actively undermines structural connectivity. The combined effects of excitotoxicity, inflammatory mediators, and oxidative stress induce cytoskeletal degradation and energy failure within axons, leading to pathological processes such as axonal swelling, disconnection, and ultimately Wallerian degeneration, which severs critical long-distance neural pathways [68,69]. Furthermore, the inflammatory cascade actively orchestrates the establishment of potent extrinsic barriers to regeneration. It drives the transformation of astrocytes into a reactive state, leading to the formation of a dense glial scar, and upregulates the expression of growth-inhibitory molecules, most notably chondroitin sulfate proteoglycans (CSPGs) in the scar core. These CSPGs, such as neurocan and versican, directly inhibit axonal growth cone advancement and collapse via specific receptor-mediated signaling pathways, creating a non-permissive terrain for rewiring [31,32,33,69,70,71].

The cumulative result of this cascade—significant loss of neurons and supporting glia, widespread disconnection of axonal pathways, and the erection of active chemical and physical barriers to plasticity—fundamentally compromises the brain’s innate capacity for neural circuit reconstruction. This multifaceted pathophysiology explains why historically, therapeutic strategies targeting single inflammatory pathways (e.g., systemic TNF-α inhibition with agents like dexanabinol, or broad immunosuppression with steroids) have failed in clinical trials to improve functional outcomes after TBI, and in some cases have shown harm [6,72,73,74,75]. These failures underscore that merely suppressing one aspect of inflammation is insufficient against such an integrated pathological network. This failure to re-establish functional networks is directly correlated with the persistence of neurological and cognitive deficits in patients, underscoring that overcoming this integrated pathophysiology is the central challenge for regenerative therapies [38,57,76].

## 3. Classification of Innovative Biomaterials and Their Mechanisms in Modulating Neuroinflammation

The limitations outlined above converge on a central conclusion: systemic pharmacological approaches aimed solely at suppressing inflammation are insufficient to promote neural repair after TBI. Successful recovery requires a coordinated multi-pronged strategy that must concurrently mitigate the hostile inflammatory milieu, provide sustained neurotrophic support, guide axonal regeneration, potentially replace lost cells, and restore vasculature. This need for multi-target, temporally coordinated intervention presents a formidable challenge for conventional Drug Deliv., which struggles with off-target effects, poor CNS bioavailability, and an inability to spatially and temporally control the presentation of therapeutic cues [6,72,73,74,75].

This fundamental therapeutic gap provides the compelling rationale for biomaterial-based platforms. Advanced biomaterials are not merely passive drug carriers; they are dynamic systems engineered to actively interface with and remodel the pathological microenvironment. They enable the localized, sustained, and potentially sequential delivery of therapeutic agents—from small molecules and proteins to cells and genetic material—directly to the lesion site. This approach minimizes systemic toxicity while allowing for integrated combination therapies. By addressing the multifaceted nature of the post-TBI pathophysiology in a spatially and temporally controlled manner, biomaterial strategies represent a paradigm shift towards precision medicine in neurotrauma [6,11,64,73,77].

In this chapter, we systematically classify and evaluate biomaterial-based therapeutic strategies for TBI. It is organized according to the primary material categories actively pursued in current research, analyzing each platform’s core mechanisms of action, key advantages, and primary challenges from a translational perspective. The discussion progresses from established material classes toward more complex and emergent designs. Specifically, the chapter consists of five main sections: (1) Natural Biomaterials, (2) Synthetic Biomaterials, (3) Nanobiomaterials, (4) Composite Biomaterials, and (5) Intelligent Biomaterials. A comparative summary of their characteristics and translational potential is provided in (Table 1).

### 3.1. Natural Biomaterials

Natural biomaterials, owing to their inherent biocompatibility, biodegradability, and diverse immunomodulatory properties, represent a promising class of therapeutic agents for mitigating the detrimental neuroinflammatory response following TBI. Among these, collagen and chitosan have garnered significant attention for their multifaceted roles in modulating the post-TBI neuroinflammatory milieu and promoting neural repair.

Collagen-based scaffolds, beyond their well-established role in providing structural support for tissue regeneration, create a bioactive microenvironment that actively modulates immune cell behavior. As demonstrated in a controlled cortical impact (CCI) model in canines, these scaffolds can favorably influence microglial activity, promoting a phenotypic shift from the destructive M1 state towards the reparative and neuroprotective M2 phenotype. This shift is characterized by the upregulation of anti-inflammatory cytokines and growth factors, and a reduction in pro-inflammatory mediators like TNF-α [78,79,80]. The unique extracellular matrix provided by collagen not only guides cellular infiltration and adhesion but also presents specific binding sites that can influence immune cell signaling, thereby fostering an environment conducive to tissue repair and regeneration. The inherent biocompatibility and low immunogenicity of collagen further underscore its potential as an ideal candidate for mitigating secondary injury processes and facilitating recovery following TBI.

**Figure 3:**
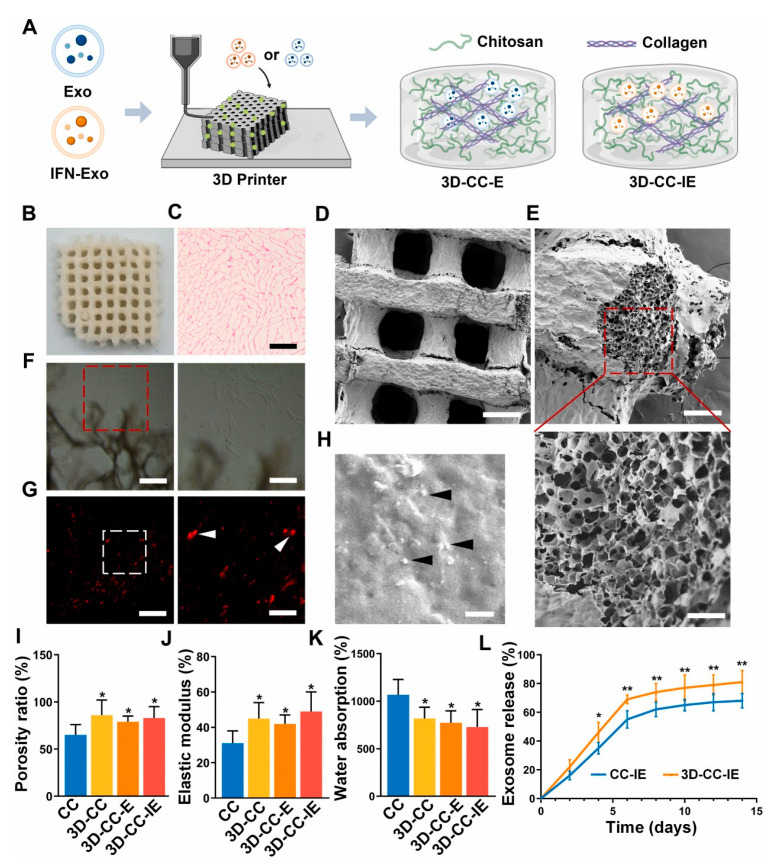
Characterization of 3D-CC-IE scaffolds. (**A**) Schematic diagram of 3D-CC-E and 3D-CC-IE scaffold preparation. (**B**–**E**) Representative images of 3D-CC-IE under general view (**B**), HE staining (**C**) and SEM (**D**,**E**). (**F**) Neural stem cells cultured on 3D-CC-IE scaffolds. (**G**) Representative 3D fluorescent image illustrated the distribution of exosomes within the 3D-CC-IE. (**H**) The distribution of exosomes within the 3D-CC-IE scaffold. (**I**–**K**) The porosity ratio (**I**), elastic modulus (**J**), and water absorption (**K**) of the scaffolds. (**L**) Cumulative release profile of IFN-Exo from the CC-IE and 3D-CC-IE within 14 days. All data were expressed as mean ± SD; * *p* < 0.05, ** *p* < 0.01 vs. CC or CC-IE. Scale bars: 50 μm in (**C**,**G**,**H**), 200 μm in (**D**), 100 μm in ((**E**) top), 20 μm in ((**E**) bottom), 500 nm in (**F**). 3D-CC-IE, 3D-printed collagen/chitosan scaffolds integrated with interferon-gamma-enhanced exosomes; 3D-CC-E, 3D-printed collagen/chitosan scaffolds integrated with exosomes; IFN-Exo, interferon-gamma-enhanced exosome; CC-IE, collagen-chitosan scaffolds integrated with interferon-gamma-enhanced exosomes; CC, collagen-chitosan scaffolds; HE staining, hematoxylin-eosin staining; SEM, scanning electron microscope [81]. Copyright (2024) ELSEVIER.

Chitosan and its derivatives exhibit remarkable capabilities in directly attenuating oxidative stress and safeguarding endothelial integrity, thereby contributing to the repair of the compromised BBB (Figure 3) [81,82,83]. As shown in a CCI model in rats, this protective effect is crucial as BBB disruption exacerbates neuroinflammation by allowing the infiltration of peripheral immune cells and harmful substances into the central nervous system [81,84]. Furthermore, chitosan has been extensively demonstrated to modulate microglial activity. Specifically, it can suppress the polarization of microglia towards the detrimental pro-inflammatory M1 phenotype by inhibiting key signaling pathways, such as the NF-κB pathway [81,84]. This inhibition leads to a significant reduction in the release of pro-inflammatory cytokines, including TNF-α, IL-1β, and IL-6, which are major contributors to secondary brain injury. Beyond direct cellular modulation, chitosan can also act as an adsorbent for inflammatory mediators, effectively reducing their local concentration and thus dampening the inflammatory cascade.

The therapeutic potential of collagen and chitosan stems from their multifunctional bioactivity, which supports complex repair processes. However, their mechanisms, often framed within the limiting M1/M2 paradigm, may not fully capture the nuanced human immune response in vivo. Furthermore, the inherent batch-to-batch variability of natural polymers poses challenges for reproducibility and predictable degradation. While generally biocompatible, their immunogenicity and the effects of their degradation products in the inflamed CNS require careful evaluation [76,80,81,85]. Thus, advancing these materials necessitates moving beyond oversimplified biological models and engineering greater precision and consistency into their design.

### 3.2. Synthetic Biomaterials

Synthetic biomaterials, such as polylactic acid (PLA) and polyvinyl alcohol (PVA), offer distinct advantages in modulating neuroinflammation and promoting recovery after TBI due to their tunable properties, controlled degradation, and versatility as Drug Deliv. platforms. Their anti-inflammatory and immunomodulatory effects are exerted through multiple sophisticated mechanisms.

**Figure 4:**
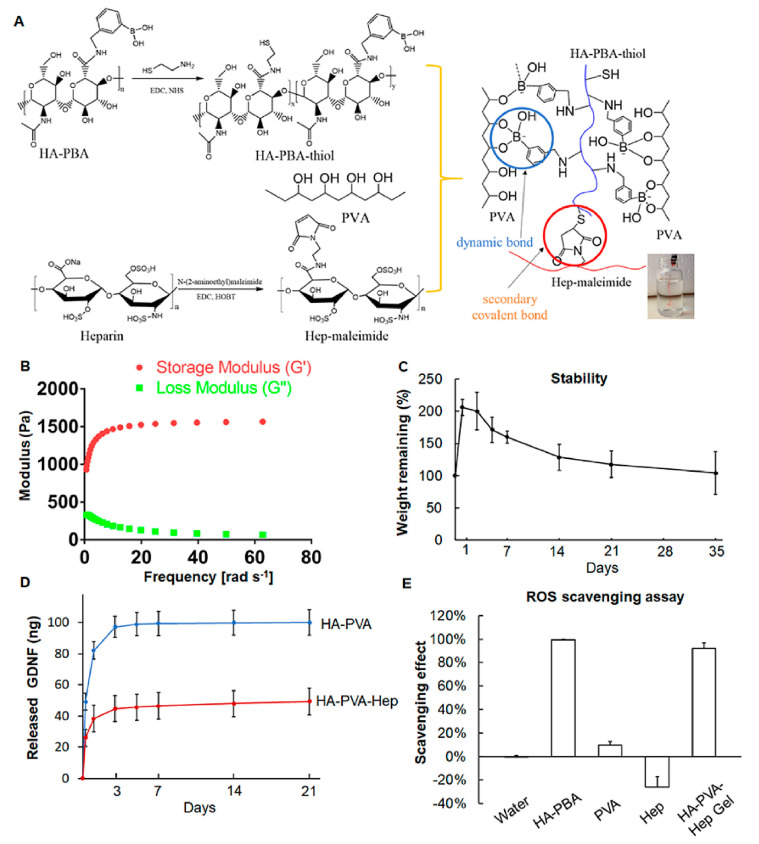
Synthesis and characterization of the HA-PVA-Hep hydrogel: (**A**) HA-PBA-thiol and Hep-maleimide were synthesized individually and then mixed together with PVA to fabricate the injectable hydrogel with dynamic boronic ester bonds and stabilized with secondary covalent bonds; (**B**) Frequency sweep testing of the hydrogel indicated that the storage modulus (G′) was consistently higher than the loss modulus (G″); (**C**) Degradation profile of the hydrogel in PBS over 35 days; (**D**) Release profile of 100 ng of GDNF from the HA-PVA hydrogel either with or without conjugated heparin for 21 days; (**E**) ROS scavenging property of the hydrogel and its precursors, including HA-PBA, PVA, and Heparin, determined by pyrogallol assay [87]. Copyright (2020) Royal Society of Chemistry.

Polylactic Acid (PLA), a biodegradable polyester, primarily exerts its immunomodulatory effects through its degradation products. Upon hydrolysis, PLA releases lactic acid, which is far more than a simple metabolic byproduct; it acts as an active immunomodulator. Lactic acid has been demonstrated to signal through the G protein-coupled receptor HCAR1 (hydroxycarboxylic acid receptor 1), also known as GPR81, expressed on various immune cells, particularly microglia. This signaling pathway plays a crucial role in metabolic reprogramming and immune cell phenotype switching. By activating HCAR1, lactic acid promotes a phenotypic shift in microglia from the detrimental pro-inflammatory M1 phenotype towards the reparative and neuroprotective M2 phenotype. This M1-to-M2 polarization is characterized by a reduction in pro-inflammatory cytokine secretion (e.g., TNF-α, IL-1β) and an increase in anti-inflammatory mediators and growth factors (e.g., IL-10, TGF-β, BDNF), thereby indirectly but significantly alleviating inflammation and fostering tissue repair. Moreover, the localized release of lactic acid can subtly modulate the pH of the microenvironment, which itself can influence immune cell activity and enzyme kinetics involved in inflammatory processes (Figure 4) [86,87,88].

Polyvinyl Alcohol (PVA), often formulated into hydrogels, demonstrates direct anti-inflammatory capabilities. PVA-based hydrogels have been shown to effectively attenuate the infiltration of inflammatory cells into the injury site. This direct action helps to limit the acute inflammatory cascade that contributes significantly to secondary brain damage [86,87]. Furthermore, PVA hydrogels can suppress astrocyte activation, a critical process in the formation of glial scars. Glial scars, while initially protective, can become inhibitory barriers to axonal regeneration and neural repair. By reducing astrocyte activation and subsequent glial scar formation, PVA contributes to creating a more permissive environment for neural recovery. The physical presence and mechanical properties of PVA hydrogels can also influence the local cellular environment, potentially dampening mechanosensitive inflammatory pathways [20,88].

**Figure 5:**
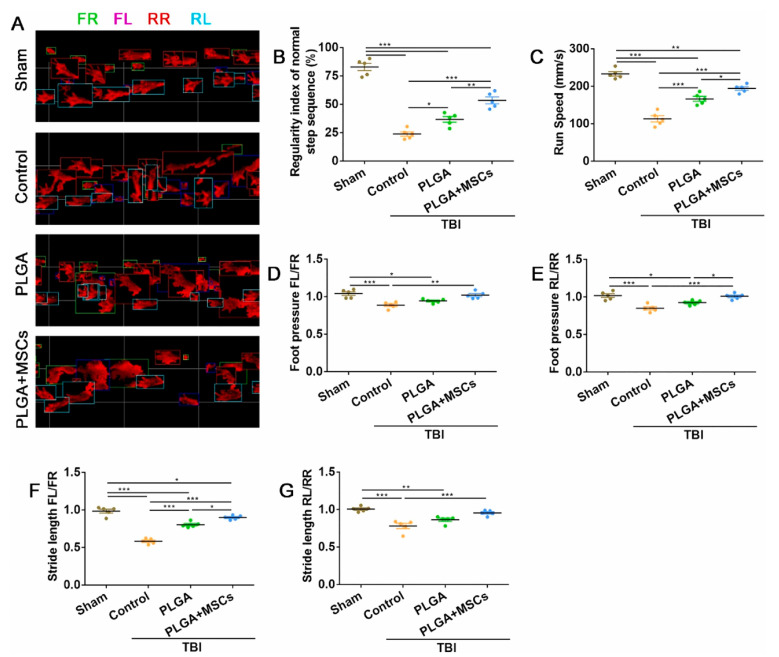
Neurological function assessed using Gait analysis. (**A**) Representative paw prints of the rats in different groups. (**B**–**G**) Gait data of each group was analyzed using GAIT SCAN analysis software. The parameters assessed included the regularity index of normal step sequence, run speed, foot pressure, and stride length. FL, FR, RL and RR, respectively, represent front left, front right, rear left and rear right paws. The data points represent individuals. The bars depict means, and the error bars represent standard error of the mean. * *p*  <  0.05, ** *p*  <  0.01, and *** *p*  <  0.001 [92]. Copyright (2024) ELSEVIER.

Beyond their intrinsic immunomodulatory properties, both PLA (frequently used in its copolymer form, poly (lactic-co-glycolic acid) or PLGA) and PVA are extensively utilized as advanced Drug Deliv. carriers. They can be precisely engineered into various nanostructures, such as nanoparticles, or macroscopic forms like hydrogels. As demonstrated in a CCI model in rats, this engineering allows for the controlled and targeted delivery of a wide array of anti-inflammatory drugs, including corticosteroids like dexamethasone, or other therapeutic agents (Figure 5) [89,90,91,92].

Synthetic biomaterials like PLA and PVA provide exceptional tunability and controlled delivery for neuroinflammation modulation. However, their proposed mechanisms warrant scrutiny. PLA’s immunomodulation via lactic acid and HCAR1 signaling relies heavily on the M1/M2 paradigm, an in vivo oversimplification. Additionally, its degradation must be carefully managed to avoid local acidification exacerbating injury. While PVA shows direct anti-inflammatory and anti-scarring potential, its long-term biocompatibility and risk of foreign body response due to mechanical mismatch require rigorous evaluation [11,36,83,86,87,93]. Critically, these polymers are not inert; their intrinsic bioactivity and degradation byproducts must be integral to therapeutic design. Future progress depends on leveraging their tunability to engage the immune system in a more nuanced, context-dependent fashion.

### 3.3. Nanobiomaterials

Nanobiomaterials, such as nano-hydroxyapatite (nHA) and mesoporous silica nanoparticles (MSNs), are emerging as highly promising therapeutic agents for modulating the hostile microenvironment that follows TBI [12,94,95,96]. An overview of common nanotherapeutic approaches for TBI is shown in (Table 2) [95].Their effectiveness primarily stems from their exceptional ability to scavenge ROS through distinct and efficient mechanisms.

Specifically, nHA demonstrates intrinsic catalase-like activity, allowing it to efficiently decompose hydrogen peroxide into water and oxygen, thereby directly neutralizing a significant ROS contributor. Meanwhile, MSNs, with their high surface area and porous structure, physically adsorb a broad spectrum of ROS, including highly reactive hydroxyl radicals and superoxide anions, effectively reducing oxidative stress [12,94,97]. The nanoscale size of these materials is crucial, enabling them to cross biological barriers, such as the blood–brain barrier, and directly interact with immune cells within the central nervous system.

A key therapeutic action is their influence on microglial polarization; for instance, surface-functionalized silica nanoparticles have been shown to suppress the pro-inflammatory NF-κB pathway and activate the anti-inflammatory Nrf2 pathway. This action shifts microglia from a pro-inflammatory M1 phenotype towards a reparative M2 phenotype, which is vital for restoring immune homeostasis and attenuating chronic neuroinflammation [98]. Beyond their intrinsic bioactivity, these nanomaterials are highly versatile as multifunctional delivery platforms. They can be engineered to concurrently load and release various therapeutic agents, such as anti-inflammatory drugs (e.g., dexamethasone) or neurotrophic factors. As shown in a weight drop injury (WDI) model in rats, this capability allows for a synergistic combination of their inherent therapeutic effects with targeted Drug Deliv. to the injury site, significantly enhancing regenerative outcomes and improving the overall therapeutic efficacy in TBI treatment [96,97,98].

Nanobiomaterials provide unique advantages for microenvironment repair through mechanisms like intrinsic ROS scavenging and pathway modulation. However, their therapeutic rationale often depends on the contested M1/M2 framework for microglial reprogramming. Significant translational challenges remain regarding their long-term fate, including potential incomplete degradation, accumulation, and unforeseen immunogenicity. The complexity of engineering them as multifunctional carriers also poses hurdles in controlling precise, synergistic release kinetics [12,82,95,99,100,101]. Thus, advancing their clinical translation requires rigorous long-term safety studies and designs that engage the immune system with greater sophistication than binary phenotypic switching.

**Table 2:** Overview of nanotherapeutic approaches for TBI with key features and therapeutic strategies. BBB: blood-brain-barrier ECM: Extracellular matrix. CSPGs: Chondroitin sulfate proteoglycan. ROS: Reactive oxygen species. RNS: Reactive nitrogen species. PAMAM: Poly(amidoamine). LNP: Lipid nanoparticle [95]. Copyright (2025) BMC.

Materials	Key Features and Therapeutic Strategy	Applications
PEGylated-polystyrene nanoparticles [102]	- Passive targeting via BBB disruption	Acute-phase Drug Deliv. leveraging transient BBB opening
	- PEGylation to prolong circulation time	
	- Size-dependent accumulation at the TBI site	
pSi nanoparticles [103]	- ECM-targeted delivery via CAQK peptide	Targeted delivery to the injury microenvironment
	- Binds to CSPGs overexpressed in the injured brain	
Carbon dot nanoparticles [104]	- Carbogenic nanozyme function	Long-term oxidative stress regulation and neuroprotection
	- Scavenges ROS and RNS	
	- Reduces oxidative stress and neuroinflammation	
Dendrimer nanoparticles [105]	- PAMAM dendrimer-based platform	Suppression of early neuroinflammatory response to prevent secondary injury
	- Sinomenine conjugation for anti-inflammation	
	- Targets microglia and suppresses neuroinflammation	
Lipid nanoparticles [106,107]	- CAQK peptide-functionalized, ROS-responsive LNP: ROS scavenging and Ca^2+^ overload inhibition	Acute-phase therapeutic delivery and prolonged circulation for sustained drug exposure
	- PEGylation density-optimized LNP: prolonged circulation, enhanced brain accumulation	
siRNA-based nanoparticles [108]	- PEGylated multimeric RNA nanoparticles	Gene silencing therapy to inhibit pro-inflammatory cytokine expression
	- RNAi-mediated TNF-α silencing	
	- Rolling circle transcription-based fabrication	
	- Enhanced BBB penetration and gene knockdown	

### 3.4. Composite Biomaterials

**Figure 6:**
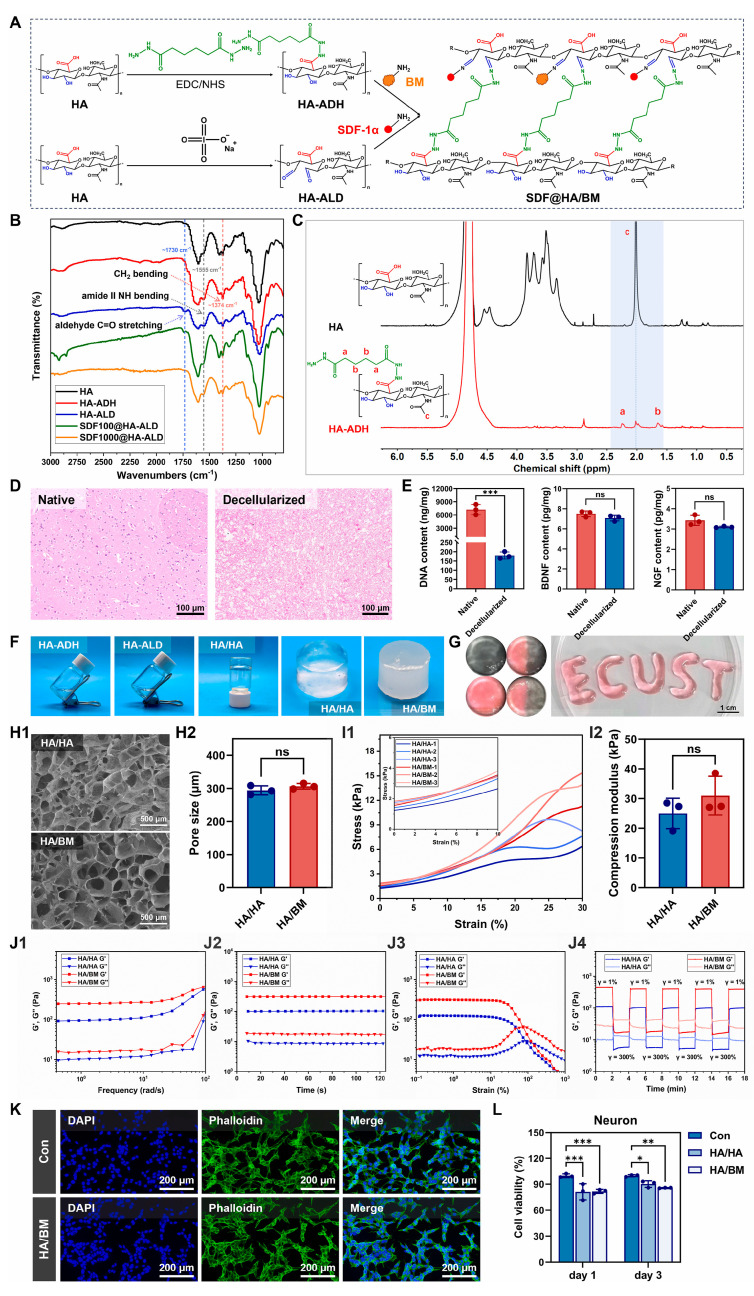
Preparation and characterization of SDF@HA/BM hydrogel. (**A**) Schematic illustration of the chemical reactions for the hydrogel preparation. (**B**) FTIR spectrums of HA, HA-ADH, HA-ALD, SDF100@HA/ALD and SDF1000@HA-ALD. (**C**) 1H NMR spectrums of HA and HA-ADH. (**D**) H&E staining images of native and decellularized brain tissue (scale bar = 100 μm). (**E**) Quantifications of DNA, BDNF and NGF in native and decellularized brains (n = 3). (**F**) The preparation diagram and appearance of two hydrogels. (**G**) Intuitive representation of self-healing and injectable properties of hydrogels (scale bar = 1 cm). (**H1**) Internal microtopography of HA/HA hydrogel and HA/BM hydrogel observed by SEM, and (**H2**) the quantification of pore sizes of two hydrogels (scale bar = 500 μm, n = 3). (**I1**) Stress–strain curves of two hydrogels under compression and (**I2**) the calculation of compression modulus (n = 3). (**J**) Rheological behaviors of HA/HA hydrogel and HA/BM hydrogel: (**J1**) the oscillatory frequency sweep curves, (**J2**) the time sweep curves, (**J3**) the amplitude sweep curves, and (**J4**) the cyclic strain sweep curves. (**K**) Cytoskeletal staining of mouse hippocampal neuronal cells (HT22) cultured with normal medium and HA/BM hydrogel (scale bar = 200 μm). (**L**) Cytocompatibility of HA/HA hydrogel and HA/BM hydrogel with neurons (n = 3). Data are expressed as mean ± SD. Two-tailed unpaired Student’s *t*-test was used for (**E**,**H2**,**I2**). Two-way ANOVA followed by Tukey’s post hoc test was used for (**L**). * *p* < 0.05, ** *p* < 0.01, and *** *p* < 0.001. ns: no significant difference. The n represents the number of independent samples, and the image-based analysis of pore size in (**H2**) is from three SEM images of three hydrogels of the same batch [109]. Copyright (2024) ELSEVIER.

Composite biomaterials, particularly polymer-ceramic systems, represent a sophisticated approach to combating neuroinflammation by synergistically combining the distinct advantages of their constituent materials. These advanced constructs, exemplified by stromal cell-derived factor-1α (SDF-1α)-loaded hyaluronic acid (HA)/decellularized brain extracellular matrix (BM) hydrogels (SDF@HA/BM) or polylactic acid-hydroxyapatite (PLA-HA) composites, are engineered to provide enhanced and multi-faceted therapeutic capabilities [109]. They achieve a potent dual anti-inflammatory effect by integrating the ROS-scavenging properties of the ceramic component (e.g., hydroxyapatite or HA) with the excellent drug-delivery capacity and structural support offered by the polymer matrix (e.g., polylactic acid or BM). This strategic combination allows for the simultaneous neutralization of harmful ROS, directly mitigating oxidative stress, and the sustained release of anti-inflammatory agents. Consequently, these materials collectively suppress the expression of key pro-inflammatory factors such as TNF-α and IL-1β. Furthermore, they play a crucial role in modulating the local immune microenvironment by influencing microglial polarization, guiding these immune cells towards a reparative M2 phenotype. As demonstrated in a CCI model in rats, this comprehensive approach, leveraging both direct ROS scavenging and the inhibition of inflammatory factor expression, offers a more robust and effective strategy for treating the complex pathology of TBI and other neuroinflammatory conditions (Figure 6) [23,109,110].

Composite biomaterials leverage synergistic properties for multi-target therapy. However, their superior functionality comes with inherent challenges. Their mechanism often relies on the contested M1/M2 framework for immune modulation. The increased material complexity raises significant manufacturing and reproducibility concerns. Furthermore, the long-term interactions between composite phases and brain tissue, including variable degradation profiles, require thorough evaluation. While promising, their clinical translation demands rigorous safety assessment and designs based on a more sophisticated understanding of immune cell function beyond phenotypic labels [11,84,94,111].

### 3.5. Intelligent Biomaterials

**Figure 7:**
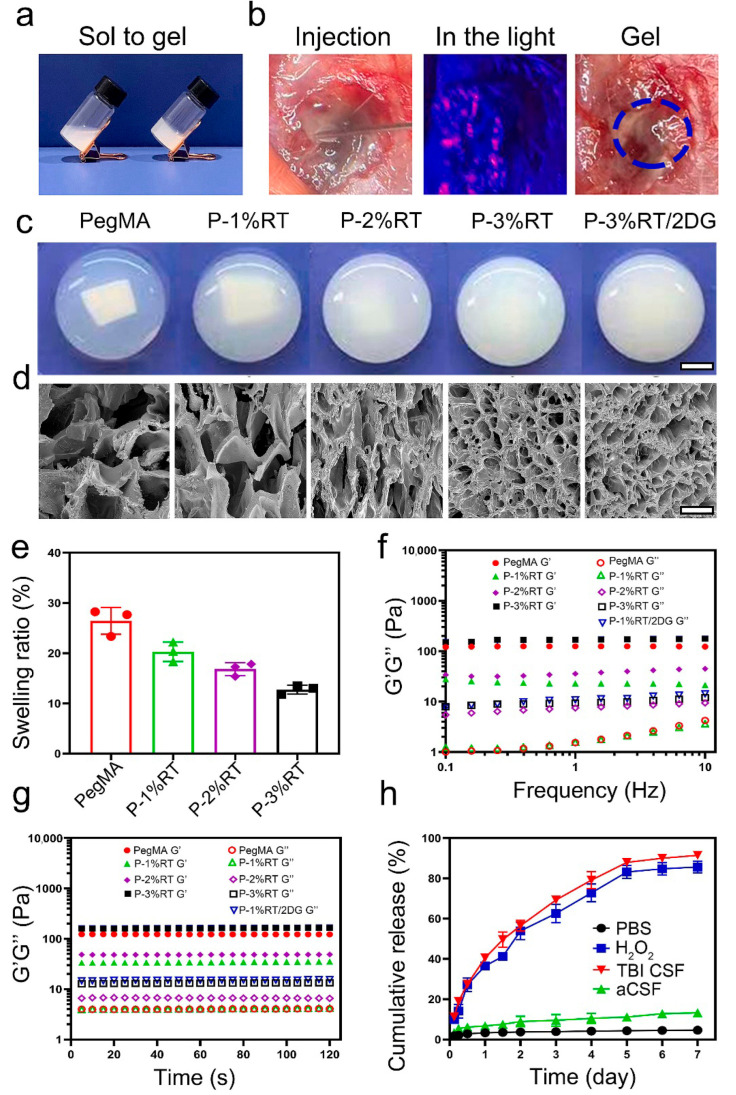
Preparation and characteristics of the hydrogel formulation. (**a**,**b**) In vitro and in vivo gelation process of the P-RT/2DG hydrogel before and after exposure to 405 nm blue light. (**c**) The macrostructure of the hydrogels with different concentrations of RT (white square tape was used to hold the hydrogel in place, scale bar: 5 mm). (**d**) Microstructures of the hydrogels with different concentrations of RT (scale bar: 200 μm). (**e**) The swelling ratio of PegMA and P-RT at different concentrations. (**f**) Frequency-sweep sequence rheological assessment of the hydrogels. (**g**) Time sweep sequence rheological test of the hydrogels. (**h**) 2DG release curves in vitro in PBS, H_2_O_2_, CSF from TBI patients and aCSF [113]. Copyright (2024) ELSEVIER.

Intelligent biomaterials, including temperature-, pH-, or ROS-responsive hydrogels, represent a significant advancement in targeted neurotherapy by enabling precise, “on-demand” anti-inflammatory action specifically at the injury site. These smart systems are meticulously engineered to remain inert under normal physiological conditions. However, upon encountering the distinct pathological signals characteristic of the traumatized brain microenvironment—such as localized acidosis (low pH), elevated levels of ROS, or temperature fluctuations—they undergo rapid physicochemical changes like swelling, dissolution, or degradation (Figure 7) [112,113].

This responsive behavior is crucial as it facilitates the controlled and triggered release of encapsulated therapeutic cargo, which can include anti-inflammatory drugs, neuroprotective peptides, or exosomes. This targeted delivery ensures that therapeutic agents are released precisely where and when they are most needed, thereby maximizing their efficacy while minimizing potential off-target side effects [57,114].

Beyond their function as sophisticated delivery vehicles, certain intelligent materials possess the capacity to directly interact with and modulate the immune microenvironment. For instance, ROS-scavenging hydrogels can directly neutralize harmful oxidants. This direct action passively reduces a primary driver of inflammation, thereby creating a more favorable milieu for tissue repair and regeneration. As shown in a WDI model in rats, this dual functionality—both as advanced delivery platforms and as direct modulators of the inflammatory response—underscores the transformative potential of intelligent biomaterials in addressing complex neuroinflammatory conditions [93,115].

Intelligent biomaterials enable precise, on-demand therapy by responding to pathological cues—a powerful concept for localized treatment. However, key challenges hinder translation. The in vivo specificity and consistency of environmental triggers like pH or ROS are limited, risking unreliable activation. Engineering such responsive systems also raises concerns about manufacturing complexity, long-term stability, and the biocompatibility of rapid degradation byproducts in neural tissue. While their direct bioactive functions (e.g., ROS scavenging) are beneficial, they do not fully address the complexity of immune dysregulation. Ultimately, advancing these materials requires designs informed by the dynamic, interconnected signaling networks of TBI, moving beyond simplified environmental triggers [113,116].

## 4. Biomaterial-Based Strategies for Promoting Neural Repair and Regeneration

### 4.1. Constructing Neural Regeneration Scaffolds

The construction of biomaterial-based scaffolds is a critical strategy for providing structural support and directional guidance to facilitate neural regeneration following TBI. This approach primarily encompasses two complementary designs: volumetric repair scaffolds and axonal guidance conduits. Volumetric repair scaffolds, often fabricated via 3D printing technologies using biomimetic materials like polycaprolactone/hydroxyapatite composites, are engineered to precisely fill the tissue voids resulting from traumatic contusions, thereby providing a physical matrix to bridge the lesion cavity and mitigate tissue collapse [84,85,110]. Concurrently, axonal guidance conduits leverage advanced techniques such as electrospinning to create highly aligned nanofibrous architectures from materials including silk fibroin/PLA, chitosan/PLA composites, and graphene/gelatin; these constructs effectively mimic the topographical cues of the native nerve sheath’s extracellular matrix, providing contact guidance for directed axonal regrowth and facilitating the re-establishment of neural circuitry across the injury site [100,117,118,119].

#### 4.1.1. Volumetric Repair Scaffolds

Volumetric repair scaffolds are crucial for addressing the significant tissue loss and subsequent cavity formation characteristic of severe TBI. These three-dimensional structures, often fabricated using advanced manufacturing techniques like 3D printing, are designed to precisely match the geometry of the brain injury. Their primary function is to provide immediate structural support, thereby preventing the collapse of surrounding tissues and creating a favorable environment for regeneration [81,110]. For instance, composite systems of polycaprolactone (PCL) and hydroxyapatite (HA) are typical materials for volumetric repair scaffolds. PCL, as a synthetic biomaterial, offers tunable biodegradability and mechanical strength, which are essential for maintaining scaffold integrity during the healing process. Hydroxyapatite, a ceramic component, enhances bioactivity, promotes cell adhesion, and may modulate local inflammatory responses, thus creating a more conducive environment for regeneration [120,121,122]. By filling the cavities caused by injury, these biomimetic scaffolds act as temporary extracellular matrix (ECM) analogs. This role is vital for promoting the infiltration of endogenous neural progenitor cells, angiogenesis (new blood vessel formation is crucial for nutrient supply), and the organized regeneration of axons. Ultimately, these scaffolds aim to restore the structural continuity and functional capacity of the damaged brain regions [84,85].

#### 4.1.2. Axonal Guidance Conduits

Axonal guidance conduits are specifically designed to meet the critical need for directed neural regeneration through electrospun nanofiber scaffolds, which topologically mimic the aligned structure of the natural neural sheath extracellular matrix. This biomimetic design is essential for providing crucial contact guidance cues that direct axonal growth cones, thereby promoting long-distance regeneration across the injury site [117]. Commonly used materials include blends of natural biomaterial silk fibroin and synthetic biomaterial polylactic acid (PLA), which combine excellent biocompatibility with tunable degradation kinetics, ensuring the scaffold’s sustained function during regeneration and subsequent degradation after its mission is complete. Furthermore, composite materials like chitosan/PLA composites leverage the inherent bioactivity of chitosan to enhance cell-scaffold interactions [123]. Beyond passive guidance, advanced conduits can also integrate conductive materials such as nanobiomaterial graphene into a gelatin matrix to form composite materials. These sophisticated structures not only provide physical guidance but also enable electrical stimulation. Electrical stimulation has been shown to further promote neurite outgrowth and enhance synaptic integration, offering an additional therapeutic dimension. By replicating the anisotropic structure of neural tissue, these nanofiber conduits act as guiding bridges. They direct axonal regeneration in a directional manner, which is critical for the successful reconstruction of functional neural pathways after TBI, ultimately aiming to restore lost neurological function [100,118,119].

### 4.2. Precise Delivery of Key Regenerative Factors

The precise delivery of therapeutic agents is paramount for effective neural repair, achieved through advanced biomaterial systems that ensure spatiotemporal control over the release of regenerative factors. Sustained release systems, utilizing microspheres and hydrogels, provide controlled, long-term delivery of essential neurotrophic factors such as BDNF, GDNF, VEGF, and NGF, maintaining their bioactivity and therapeutic concentration at the injury site to promote neuronal survival, axonal elongation, and angiogenesis [56,111,124,125]. Complementing this, targeted delivery strategies for stem cells and exosomes enhance their therapeutic potential; for instance, stem cell transplantation is augmented by combination with piezoelectric materials, where mechanical stimulation promotes cell integration and differentiation, while MSC-derived exosomes—key modulators of neuroregenerative pathways like PI3K/Akt—are encapsulated in degradable hydrogels for localized release or loaded onto magnetic nanocarriers to improve their bioavailability and blood–brain barrier penetration efficiency through external magnetic guidance [15,100,126].

#### 4.2.1. Sustained Release Systems for Neurotrophic Factors

Sustained release systems utilizing microspheres and hydrogels are critically important for overcoming the short half-life and rapid degradation of key neurotrophic factors like Brain-Derived Neurotrophic Factor (BDNF), Glial Cell Line-Derived Neurotrophic Factor (GDNF), Vascular Endothelial Growth Factor (VEGF), and Nerve Growth Factor (NGF). These biomaterial-based carriers are engineered to provide controlled, long-term release, maintaining therapeutic concentrations directly at the injury site. For instance, biodegradable polymeric microspheres, such as those made from chitosan, encapsulate the factors and release them gradually as the polymer degrades, effectively prolonging their activity [125,127]. Similarly, injectable hydrogels, formed from natural or synthetic polymers, can be loaded with these proteins and form a depot upon injection, allowing for sustained diffusion-based release [56,111,124,128]. This localized and prolonged delivery paradigm supports crucial regenerative processes, including neuronal survival, axonal elongation, synaptogenesis, and the promotion of new blood vessel formation (angiogenesis), which are essential for reconstructing the damaged neural circuitry after TBI [129,130].

#### 4.2.2. Targeted Delivery of Stem Cells and Exosomes

Stem cells and exosomes have garnered significant attention due to their potent neuroprotective and regenerative potential. Biomaterials can provide a protective microenvironment for these biological entities and guide their survival, proliferation, and differentiation at the injury site [16,131,132,133]. For stem cell delivery, hydrogel scaffolds made from natural biomaterials (e.g., collagen, chitosan) or synthetic biomaterials (e.g., polyethylene glycol) can serve as carriers for stem cells, providing a three-dimensional growth environment and mimicking the mechanical and biochemical signals of the natural extracellular matrix, thereby promoting stem cell engraftment and neural differentiation [17,100,126,132,134]. For exosome delivery, exosomes, as carriers of intercellular information, are rich in proteins, lipids, and nucleic acids, and play roles in modulating neuroinflammation, promoting angiogenesis, and neural regeneration [48,135,136,137,138]. To enhance the stability and targeting of exosomes, researchers have developed various nanobiomaterial delivery systems, such as magnetic nanocarriers, which can enhance the ability of exosomes to penetrate the blood–brain barrier and achieve magnetic field-guided targeted delivery. Additionally, composite piezoelectric materials and degradable hydrogels are also used for exosome encapsulation and release, enabling on-demand release through external stimuli (e.g., ultrasound) to further improve therapeutic efficacy [15,17,76,126].

### 4.3. Remodeling the Neural Regenerative Microenvironment

Beyond providing structural support and delivering therapeutic factors, biomaterials play a crucial role in actively remodeling the neural regenerative microenvironment [53,54,130]. This involves simulating the extracellular matrix, regulating immune responses, and controlling blood–brain barrier repair and glial scar formation to create an optimal environment for neurorepair and regeneration.

#### 4.3.1. Simulating the Extracellular Matrix

The extracellular matrix (ECM) in the central nervous system (CNS) is a complex network of proteins and carbohydrates that provides structural support, regulates cell behavior, and influences neural development and plasticity [61]. After TBI, the ECM is often disrupted, leading to an unfavorable environment for regeneration. Biomaterials can be engineered to mimic the biochemical and biophysical properties of the natural ECM, thereby promoting neural cell adhesion, migration, proliferation, and differentiation. For instance, hydrogels composed of natural polymers like hyaluronic acid, collagen, or fibrin, or synthetic polymers like polyethylene glycol (PEG), can be functionalized with specific peptides (e.g., RGD sequences) that bind to cell surface receptors, enhancing cell-material interactions [139]. These biomimetic scaffolds can guide neurite outgrowth and synaptic formation, facilitating the reconstruction of neural circuits. Furthermore, the mechanical properties of these biomaterials, such as stiffness and elasticity, can be tuned to match those of native brain tissue, which is crucial for regulating stem cell fate and promoting neuronal differentiation.

#### 4.3.2. Regulating Immunity

Neuroinflammation is a critical component of the secondary injury cascade following TBI, contributing to neuronal damage and inhibiting regeneration. Biomaterials can be designed to modulate the immune response, shifting the microenvironment from pro-inflammatory to pro-regenerative [60]. This can be achieved through several mechanisms. Some biomaterials possess intrinsic immunomodulatory properties; for example, chitosan, a natural polysaccharide, has been shown to reduce pro-inflammatory cytokine production and promote the polarization of microglia/macrophages towards an anti-inflammatory, pro-regenerative phenotype (M2 phenotype). Another approach involves the controlled release of immunomodulatory agents, where biomaterial-based delivery systems can encapsulate and release anti-inflammatory drugs (e.g., corticosteroids, NSAIDs), immunosuppressants, or immunomodulatory cytokines (e.g., IL-10, TGF-β). This targeted and sustained release at the injury site can effectively suppress detrimental inflammation while promoting beneficial immune responses that support tissue repair. Additionally, TBI often leads to an increase in ROS, contributing to oxidative stress and inflammation. Biomaterials with antioxidant properties, such as those incorporating cerium oxide nanoparticles or selenium, can scavenge ROS, thereby mitigate oxidative damage and reduce inflammatory signaling. By actively regulating the immune microenvironment, biomaterials help to create a more permissive environment for neural survival and regeneration, reducing secondary injury and enhancing functional recovery.

#### 4.3.3. Blood–Brain Barrier Repair/Glial Scar Control

The disruption of the BBB and the formation of a dense glial scar are major impediments to neural regeneration after TBI. Biomaterials offer promising strategies to address these challenges. For BBB repair, biomaterials can be designed to promote the integrity of the BBB; for example, hydrogels containing growth factors like VEGF or angiopoietin-1 can stimulate angiogenesis and strengthen endothelial cell junctions, thereby restoring BBB function and preventing the infiltration of harmful substances and immune cells into the brain parenchyma [27,46,140]. Some biomaterials can also act as temporary physical barriers to reduce leakage across the compromised BBB. Regarding glial scar modulation, the glial scar, primarily formed by reactive astrocytes, acts as a physical and chemical barrier to axonal regrowth. Biomaterials can be engineered to modulate glial scar formation by inhibiting astrocyte reactivity, where they release factors that suppress astrocyte activation and proliferation, reducing the deposition of inhibitory molecules within the scar. Furthermore, some biomaterials can be loaded with enzymes (e.g., chondroitinase ABC) that degrade inhibitory components of the glial scar, such as chondroitin sulfate proteoglycans (CSPGs), thereby creating a more permissive environment for axonal regeneration. Lastly, biomaterials can offer alternative, permissive substrates for axonal growth that can bridge the glial scar, guiding axons through or around the inhibitory region. By simultaneously addressing BBB integrity and glial scar formation, biomaterials can significantly improve the chances of successful axonal regeneration and functional recovery after TBI [26,141,142,143,144,145].

## 5. Challenges and Opportunities in Translation from Bench to Bedside

**Figure 8:**
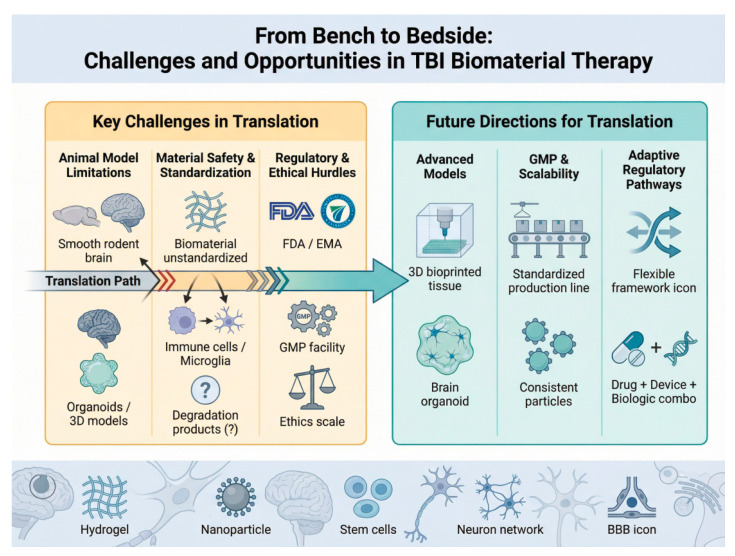
Challenges and opportunities in translation in TBI biomaterial therapy from bench to bedside.

The promising preclinical outcomes of biomaterial-based strategies for TBI underscore their significant therapeutic potential. However, the journey from laboratory validation to successful clinical application is fraught with multifaceted challenges. A critical and systematic examination of these translational barriers, coupled with a clear delineation of future opportunities, is essential for advancing the field (Figure 8).

### 5.1. Limitations of Animal Models

Based on the extensive literature reviewed in this manuscript, the most frequently employed animal models of TBI in biomaterial research are the controlled cortical impact (CCI), weight-drop (e.g., Feeney or Marmarou models), and fluid percussion injury (FPI, particularly lateral FPI) models. These models are favored for their well-characterized injury mechanisms, high reproducibility, and the ability to generate scalable injuries that mimic key aspects of human TBI pathology [146,147]. The choice of model often depends on the specific research focus: CCI is excellent for creating precise, focal cortical contusions relevant to studying localized repair; weight-drop models are versatile for generating diffuse axonal injury; and lateral FPI effectively replicates mixed focal and diffuse injuries with significant secondary pathophysiology, making it a common platform for evaluating neuroprotective and regenerative strategies [148,149].

The predominance of these models provides a consistent preclinical framework but also underscores the need to critically assess their translational relevance. A significant translational hurdle for biomaterial-based therapies lies in the inherent limitations of prevailing preclinical models, which inadequately capture the heterogeneity of human TBI [146,150,151]. Most foundational studies are conducted in young, healthy, genetically identical rodents of a single sex, under highly controlled conditions. This stands in stark contrast to the human TBI population, which exhibits vast diversity in age, pre-existing comorbidities, genetic background, and sex—all factors known to significantly influence neuroinflammatory responses and recovery trajectories.

Consequently, therapeutic mechanisms defined within these constrained models, such as the precise M1-to-M2 microglial polarization often reported for biomaterial interventions, may not reliably translate to the complex and variable immune landscape of human patients. While rodent models remain invaluable for initial proof-of-concept and safety testing [152,153,154], this paradigm gap means that strategies showing robust efficacy in the lab may fail in clinical trials due to unanticipated pathophysiological differences or a lack of effect on clinically relevant functional outcomes. Therefore, there is a pressing need to advance preclinical research through more sophisticated models, such as aged or diseased animals, organoids, and large-animal models that better mimic human neuroanatomy and immune function, to improve predictive validity and de-risk clinical translation [75,147,155,156].

### 5.2. Material Safety and Standardization

The clinical translation of innovative biomaterials faces significant hurdles related to material safety and manufacturing standardization, which are critical for regulatory approval and eventual clinical adoption. A primary concern is potential immunogenicity, as even biocompatible materials can elicit unintended inflammatory or immune responses upon implantation in the sensitive brain environment, potentially exacerbating neuroinflammation rather than alleviating it [157]. Furthermore, batch-to-batch variability during the synthesis of complex materials, particularly natural polymers and nanoparticles, poses a major challenge to ensuring consistent composition, structure, and therapeutic performance, which is a fundamental requirement for clinical-grade products [109,110]. Perhaps most critically, there is often an incomplete understanding of long-term degradation metabolites; while a material itself may be biocompatible, the biological effects of its breakdown products over months or years within the cranial cavity are not fully characterized, raising concerns about chronic toxicity or unforeseen neurological consequences [61]. Addressing these challenges requires rigorous biocompatibility profiling according to ISO 10993 standards [158], the implementation of Good Manufacturing Practice (GMP) to ensure batch consistency, and the development of advanced analytical methods to thoroughly trace the fate of materials in vivo [23,111,159,160].

### 5.3. Regulatory and Ethical Hurdles

The path to clinical application for advanced biomaterial-based therapies, particularly complex products like exosome-biomaterial combinations, is fraught with regulatory and ethical hurdles for which standardized frameworks are still evolving [15,76,134]. The primary regulatory challenge lies in the classification and quality control of these hybrid products; it is often unclear whether they should be regulated as medical devices, combination products, or biologic drugs, as they possess characteristics of all three. This ambiguity complicates the establishment of clear benchmarks for sterility, potency, purity, and stability. Specifically for exosome-biomaterial systems, standardization is challenging due to the inherent heterogeneity of exosomes derived from stem cells and the potential for batch-to-batch variation in the biomaterial scaffold. Ethically, the use of stem cell-derived components raises questions regarding donor consent and the long-term tracking of safety outcomes. Furthermore, the high cost and complexity of manufacturing these sophisticated therapies under Good Manufacturing Practice (GMP) conditions pose significant barriers to commercialization and equitable access. Overcoming these obstacles requires proactive dialogue between researchers, regulatory agencies (such as the FDA and EMA), and ethicists to develop new, adaptive regulatory pathways that can ensure both safety and efficacy without stifling innovation [99,101,161].

### 5.4. Promising Biomaterial Platforms for Clinical Translation

Based on current research, several biomaterial strategies appear particularly promising. First, adaptive or intelligent biomaterials (e.g., ROS-scavenging or enzyme-responsive hydrogels) that can respond to specific pathological cues in the injury microenvironment offer a significant advantage for spatiotemporally controlled drug release, maximizing local efficacy while minimizing systemic side effects [112,113]. Second, multifunctional platforms that integrate multiple therapeutic actions—such as combining anti-inflammatory, antioxidant, and pro-regenerative signals within a single scaffold—are highly compelling, as they address the synergistic nature of post-TBI damage mechanisms. Promising examples include composite hydrogels delivering both immunomodulatory drugs and neurotrophic factors, or nano-biomaterial hybrids designed for sequential release [23,109,110]. Third, advancements in natural polymer-based materials (e.g., hyaluronic acid, collagen) and bioinspired synthetic matrices that closely mimic the brain’s extracellular matrix show great potential due to their superior biocompatibility and ability to support host tissue integration [86,87,92]. Finally, stem cell or exosome-laden biomaterial systems, which enhance the retention and directed function of these therapeutic cells/vesicles, represent a powerful regenerative strategy moving toward clinical testing [15,77,133]. The most translatable path forward likely involves a combination of these approaches, focusing on materials that are not only effective but also scalable, reproducible, and designed with clear regulatory and clinical trial endpoints in mind [6,11,12,14].

### 5.5. Future Directions for Translation

Evaluating the clinical translation potential of biomaterials for TBI requires considering not only therapeutic efficacy in preclinical models but also key translational criteria such as biocompatibility, safety, manufacturability, and alignment with the dynamic, multi-faceted pathology of human TBI. The successful translation of innovative biomaterial-based therapies for TBI from the laboratory to the clinic hinges on a multi-faceted approach that addresses key challenges in validation, manufacturing, and regulation. Critical future directions include the development of more sophisticated, human-relevant multi-dimensional models—such as 3D-bioprinted tissues and organoids—to rigorously and predictively validate therapeutic efficacy [162,163,164]. Concurrently, advancing Good Manufacturing Practice (GMP)-compliant production processes is essential to ensure the scalability, reproducibility, and quality control of complex products, particularly exosome-biomaterial hybrid systems [77,165,166]. Finally, establishing robust, adaptive clinical evaluation and regulatory standards, with clearly defined efficacy endpoints and specialized approval pathways, is paramount for navigating these novel therapies through the development pipeline and ensuring their safe and effective application in patients [73,167].

#### 5.5.1. Developing Multi-Dimensional TBI Models to Rigorously Validate Efficacy

A critical direction for de-risking clinical translation is the development and adoption of more sophisticated, multi-dimensional TBI models that can more accurately predict therapeutic efficacy in humans [164,168]. Moving beyond conventional rodent models, future efforts should focus on integrating advanced in vitro and in silico systems. This includes utilizing human-based models such as 3D-bioprinted brain tissues, patient-derived organoids, and sophisticated microfluidic “brain-on-a-chip” platforms that can incorporate human neurons, glia, and vascular cells to recapitulate key aspects of the human neuroinflammatory and regenerative response. These systems allow for high-throughput screening of biomaterial interactions in a human-specific context [162,163,169]. Furthermore, the integration of biomaterial scaffolds within these models is essential to study how the implants interact with a complex, multi-cellular human microenvironment. Complementing this, advanced computational models can help predict biomaterial degradation and drug release kinetics in vivo. By validating biomaterial efficacy in these multi-faceted human-relevant systems, researchers can generate more robust and predictive data, thereby building a stronger foundational rationale before proceeding to costly and complex large-animal studies and human clinical trials [170,171].

#### 5.5.2. Advancing GMP-Compliant Production of Exosome-Biomaterial Hybrid Systems

A pivotal step towards the clinical realization of these advanced therapies is the establishment of robust, scalable, and Good Manufacturing Practice (GMP)-compliant production processes for exosome-biomaterial hybrid systems [77,172]. The current manufacturing pipeline faces significant challenges in standardization, including the scalable production and purification of consistent, potent exosome batches from mesenchymal stem cells (MSCs), and the reproducible integration of these exosomes with biomaterial scaffolds (e.g., hydrogels, nanoparticles) without compromising the biological activity of the exosomes or the physicochemical properties of the biomaterial [173,174,175]. Future directions must focus on developing closed, automated bioreactor systems for exosome production, implementing rigorous quality control assays for exosome characterization (e.g., tetraspanin profiling, RNA content, and functional potency assays), and defining critical quality attributes for the final combined product. Furthermore, creating standardized protocols for the sterile loading, sustained release profiling, and long-term stability testing of these hybrid systems is essential for regulatory approval [165,166,176]. Successfully navigating these challenges will require close collaboration between bioengineers, manufacturing scientists, and regulatory bodies to create a new framework that ensures the identity, purity, potency, and safety of these complex therapeutic products, thereby enabling their transition from promising lab-scale discoveries to commercially viable and clinically accessible medicines [15].

#### 5.5.3. Establishing Robust Clinical Evaluation and Regulatory Standards for These Novel Therapies

The successful clinical translation of innovative biomaterial-based therapies for TBI is critically dependent on the parallel development of robust clinical evaluation frameworks and adaptive regulatory standards tailored to their unique mechanisms of action. The inherent complexity of products such as exosome-embedded hydrogels or bioactive scaffolds necessitates a departure from conventional drug evaluation paradigms [16,73,111]. A key future direction involves the establishment of clearly defined efficacy endpoints that go beyond traditional neurological scores to incorporate advanced neuroimaging biomarkers, such as quantitative MRI metrics for tracking tissue integration and inflammation resolution, and sensitive electrophysiological measures of functional connectivity [45,177,178,179]. Regulatory agencies will need to create specialized approval pathways for these combination products, potentially leveraging adaptive trial designs and master protocols to efficiently test multiple related biomaterial platforms. Furthermore, comprehensive post-market surveillance registries will be essential for monitoring long-term safety, degradation profiles, and real-world effectiveness [167,180,181]. Achieving this requires proactive collaboration between researchers, clinicians, industry partners, and regulatory bodies like the FDA and EMA to develop consensus standards for product characterization, potency assays, and clinical trial design, thereby creating a clear and feasible pathway for these promising therapies to reach patients while ensuring rigorous evaluation of their safety and benefits.

## 6. Future Perspectives

### 6.1. Intelligent Responsive Materials

The next frontier in biomaterial-based therapy for TBI lies in the development of cutting-edge intelligent responsive materials that can actively sense dynamic pathological cues within the injury microenvironment and adapt their therapeutic actions in real-time [127,130]. These next-generation systems are engineered to react to specific local signals, such as elevated levels of ROS, acidic pH, overexpressed enzymes (e.g., matrix metalloproteinases), or aberrant electrical activity, which are hallmark features of the secondary injury phase. Upon detection, these materials undergo predictable physicochemical changes—such as degradation, swelling, or charge reversal—leading to the on-demand release of anti-inflammatory drugs, neurotrophic factors, or exosomes precisely when and where they are most needed [57,93,112,113,114,115]. This feedback-loop mechanism moves beyond static delivery, enabling a highly localized, adaptive, and potent therapeutic response that can modulate the inflammatory cascade, protect vulnerable neural tissue, and promote repair processes with unprecedented spatiotemporal control, ultimately paving the way for personalized and highly effective treatment regimens for TBI.

### 6.2. Multi-Modal Therapeutic Systems

Future therapeutic platforms will increasingly leverage multi-modal approaches that integrate complementary strategies within a single biomaterial system to achieve synergistic effects unmatched by any single modality [74]. These advanced systems are designed to concurrently address the multifaceted pathology of TBI by combining, for example, the sustained release of anti-inflammatory drugs and neuroprotective exosomes with physical stimulation techniques such as electrical stimulation and optogenetics [12]. A single scaffold could be engineered from conductive polymers to deliver tailored electrical cues that promote neuronal differentiation and axonal guidance, while its porous structure slowly releases exosomes to modulate inflammation and promote synaptic plasticity [182]. Furthermore, the incorporation of light-sensitive ion channels or actuators could enable precise, spatiotemporal control over specific neuronal populations using optogenetics, all while the biomaterial matrix itself provides critical structural support [183]. This convergence of biochemical, biophysical, and bioelectronic cues within a unified platform allows for a comprehensive and dynamically adaptable intervention, targeting neuroinflammation, cell death, and failed regeneration simultaneously, thereby significantly enhancing the potential for functional neural circuit restoration after severe TBI [74,116,184,185].

### 6.3. Personalized Medicine

The future of TBI management is poised to embrace personalized medicine, moving beyond a one-size-fits-all approach through the development of biomaterial strategies tailored to the individual patient’s specific injury characteristics and biological profile. This paradigm shift involves customizing biomaterial-based interventions based on clinical imaging data that defines the TBI subtype (e.g., focal contusion vs. diffuse axonal injury), the precise geometry of the lesion, and the patient’s unique biomarker signature, including levels of specific inflammatory cytokines, neurotrophic factors, and genetic predispositions [186,187,188]. For instance, a patient with a dominant pro-inflammatory cytokine profile (e.g., high IL-1β) might receive a hydrogel pre-loaded with a targeted anti-IL-1β antibody, while a patient with impaired neurotrophic support might benefit from a scaffold designed for sustained release of BDNF. Advanced manufacturing techniques like 3D printing and biofabrication will enable the creation of patient-specific scaffolds that perfectly match the lesion cavity, while “smart” materials with tunable properties will allow for the precise adjustment of drug release kinetics and material degradation rates to align with the patient’s dynamic healing trajectory [113,114]. This highly individualized approach, powered by diagnostic biomarkers and advanced manufacturing, promises to maximize therapeutic efficacy by ensuring the right intervention is delivered to the right patient at the right time [189,190,191].

### 6.4. Optimized Clinical Translation Pathways

A critical future perspective involves the deliberate optimization of clinical translation pathways through enhanced “bridge studies” specifically designed to strengthen the fragile link between preclinical animal validation and human applications. Recognizing the limitations of animal models, these studies will focus on generating robust, predictive data by utilizing higher-fidelity models, such as large animals with gyrencephalic brains (e.g., swine, non-human primates) that more closely mimic human TBI pathophysiology and healing responses [192]. Furthermore, a key strategy is the implementation of “reverse translation” approaches, where biomarkers and clinical observations from human TBI patients are used to inform and refine the design of animal models and efficacy endpoints, ensuring they are clinically relevant [193,194]. The establishment of multi-center, standardized preclinical trials with rigorous blinding, randomization, and statistical power will enhance the reliability and generalizability of the data. Proactive and early engagement with regulatory agencies to define clear and feasible development paths for these complex therapeutic products is equally essential. By systematically strengthening this translational bridge through large animal studies, clinically relevant endpoints, and regulatory alignment, the field can significantly de-risk the transition to first-in-human trials and accelerate the delivery of promising biomaterial-based therapies to patients in need [195,196,197,198].

## 7. Conclusions

In summary, innovative biomaterials offer a transformative, multi-faceted approach for the treatment of TBI, enabling synergistic neuroprotection and repair through integrated mechanisms that include targeted anti-inflammatory action, potent antioxidant effects, provision of critical structural support, and the advanced delivery of therapeutic agents like exosomes. These platforms effectively overcome the significant limitations of conventional therapies by providing sustained, localized intervention at the injury site. Looking forward, the future development of this field should critically focus on the deeper integration of biologically active exosome technology with next-generation smart biomaterials. This powerful combination is poised to drive a paradigm shift away from broad, non-specific interventions towards precise, personalized, and clinically viable therapies that can dynamically interact with the injury microenvironment to promote holistic and functional recovery for TBI patients.

## Figures and Tables

**Table 1:** Classification and translational potential of biomaterial platforms for TBI therapy. BBB: Blood–Brain Barrier. ROS: Reactive Oxygen Species. ECM: Extracellular Matrix. PLA: Polylactic Acid. PLGA: Poly (lactic-co-glycolic acid). PVA: Polyvinyl Alcohol. CNS: Central Nervous System. HA: Hyaluronic Acid. nHA: nano-Hydroxyapatite. MSNs: Mesoporous Silica Nanoparticles.

Biomaterial Category	Typical Examples	Key Mechanisms	Advantages	Likelihood of Near-Term Clinical Translation	Challenges for Translation
Natural Biomaterials	Collagen, Chitosan	Immunomodulation	Biocompatibility	Moderate to High	Batch-to-batch variability; oversimplified reliance on M1/M2 paradigm for mechanism; immunogenicity of degradation products in the inflamed CNS.
		BBB protection	Biodegradability		
		ECM-like scaffold	Bioactive signaling		
Synthetic Biomaterials	PLA/PLGA, PVA	Immunomodulation	Tunable properties	High (as drug carriers)	Potential local side effects (e.g., acidification from PLA); long-term biocompatibility and foreign body response; mechanisms often based on simplified M1/M2 models.
		Anti-inflammatory/scarring effects	Reproducible manufacturing		
		Tunable Drug Deliv.	Excellent capability		
Nanobiomaterials	nHA, MSNs	ROS scavenging	Passability to BBB	Moderate	Complex long-term fate (degradation, accumulation, potential immunogenicity); intricate engineering for controlled release; reliance on contested cellular phenotype (M1/M2) models.
		Pathway modulation	High surface area		
		Multifunctional Drug Deliv.	Multi-functionalization potential		
Composite Biomaterials	HA/Brain ECM Hydrogels, PLA-HA composites	Synergistic combination	Superior functionality by design	Low to Moderate (currently)	High manufacturing complexity and reproducibility concerns; variable degradation profiles of composite phases; long-term interaction with brain tissue is less predictable; often relies on simplified immune modulation concepts.
		Multi-target therapy	Address multiple pathological aspects simultaneously.		
Intelligent Biomaterials	pH/ROS/temperature-responsive hydrogels	Responsive release	Spatiotemporal precision	Low to Moderate (evolving)	Unreliable specificity and consistency of in vivo pathological triggers; manufacturing and long-term stability challenges; biocompatibility of rapid degradation byproducts; does not fully address immune dysregulation complexity alone.
		Direct bioactivity	Targeted neurotherapy		

## Data Availability

No new data were created or analyzed in this study. Data sharing is not applicable to this article.
